# Retinal vessel density from optical coherence tomography angiography to differentiate early glaucoma, pre-perimetric glaucoma and normal eyes

**DOI:** 10.1371/journal.pone.0170476

**Published:** 2017-02-02

**Authors:** Handan Akil, Alex S. Huang, Brian A. Francis, Sirinivas R. Sadda, Vikas Chopra

**Affiliations:** 1 Doheny Eye Institute, Doheny Image Reading Center, Los Angeles, CA, United States; 2 Department of Ophthalmology, David Geffen School of Medicine at UCLA, Los Angeles, CA, United States; University of Houston, UNITED STATES

## Abstract

**Purpose:**

To evaluate optic nerve vascular density using swept source optical coherence tomography angiography (OCTA) in patients with early primary open angle glaucoma (POAG), pre-perimetric glaucoma and normal eyes.

**Methods:**

This is a prospective, observational study including 56 eyes in total and divided into 3 groups; 20 eyes with mild POAG, 20 pre-perimetric glaucoma eyes, and 16 age-matched normal eyes as controls. The optic disc region was imaged by a 1050-nm-wavelength swept-source OCT system (DRI OCT Triton, TOPCON). Vessel density was assessed as the ratio of the area occupied by the vessels in 3 distinct regions: 1) within the optic nerve head; 2) in the 3 mm papillary region around the optic disc; and 3) in the peripapillary region, defined as a 700-μm-wide elliptical annulus around the disc. The potential associations between vessel density and structural, functional measures were analyzed.

**Results:**

There was a statistically significant difference for the peripapillary vessel density, optic nerve head vessel density, and papillary vessel density among all the groups (p<0.001). Control eyes showed a significant difference for all measured vessel densities compared to glaucomatous eyes (p values from 0.001 to 0.024). There was a statistically significant difference between control and pre-perimetric glaucoma eyes for peripapillary, optic nerve head and papillary vessel density values (p values from 0.001 to 0.007). The optic nerve head vessel density, superior and inferior papillary area vessel density (Pearson r = 0.512, 0.436, 0.523 respectively) were highly correlated with mean overall, superior and inferior RNFL thickness in POAG eyes (p = 0.04, p = 0.02 and p = 0.04 respectively). Multiple linear regression analysis of POAG group showed that optic nerve head vessel density in POAG group was more strongly linked to RNFL thickness than to any other variables.

**Conclusions:**

Eyes with mild POAG could be differentiated from pre-perimetric glaucoma eyes, which also could be differentiated from normal eyes using OCTA-derived retinal vessel density measurements.

## Introduction

Glaucoma is a group of eye diseases that result in damage to the optic nerve and potentially leads to irreversible blindness [1]. The nerve damage involves loss of retinal ganglion cells in a characteristic pattern [2–5] with intraocular pressure (IOP) the only modifiable risk factor and IOP reduction the only treatment. Previous studies have reported that vascular factors may play a critical role in the development of glaucoma in addition to elevated IOP [3–7].

Pre-perimetric glaucoma patients represent a group of individuals with risk factors such as elevated IOP or ocular findings such as optic disc cup enlargement that are suspicious for glaucoma, but often do not have the classic optic nerve neuroretinal rim loss or characteristic visual field defects to meet the formal definition of definite glaucoma. In terms of functional assessments, visual field (VF) testing using standard automated perimetry remains the standard criterion for glaucoma diagnosis and assessment, but it has substantial variability, often with poor reproducibility [8]. Optical coherence tomography (OCT) gives an objective measurement of retinal nerve fiber layer (RNFL) thickness and/or ganglion cell complex (GCC) which is important for glaucoma assessment but is limited utility in advanced disease and does not relate to cause of disease as opposed to the final presentation [9].

Previously, microvascular changes of optic nerve head and peripapillary area have been demonstrated in patients with glaucoma [10,11]. Recent studies suggested that another possible imaging modality OCT angiography (OCTA), may be used for early diagnosis and monitoring of glaucoma [12,13]. Although no current technology including Laser Doppler Flowmetry can provide flow rate in smaller retinal vessels, OCTA has been developed as a non-invasive imaging technique that generates three-dimensional, depth encoded images of small and large caliber retinal vasculature within the eye by using motion contrast. It is based on comparison of repeat scans acquired at the same position in the retina to look for changes in the scan of blood flow. It does mapping by A scan to A scan comparison of two or more OCT volumetric cubes which provides detailed vasculature of the retina and optic nerve head, in a noninvasive manner, using OCT scanning alone and without the use of any kind of exogenous dye needed in other vascular imaging techniques like fluorescein angiography [14]. To the best of our knowledge, there are no previous published reports that have evaluated the optic nerve head, papillary and peripapillary retinal vasculature using OCT angiography in eyes with mild POAG and compare the results with pre-perimetric glaucoma and healthy eyes. Understanding the relationship between retinal vessel density and early glaucoma may increase our understanding of the role of retinal blood flow in the glaucoma cascade and in the pathophysiology of glaucoma. The purpose of this study was to measure the retinal vessel density using OCT angiography in mild POAG eyes *vs* pre-perimetric glaucoma eyes *vs* normal eyes, and to investigate correlations between retinal vessel density measurements to other structural parameters like RNFL thickness and functional VF parameters.

## Methods

Our prospective, observational study was performed between February 2016 and May 2016 at the UCLA Doheny Eye Center glaucoma clinic. The research protocols were approved by the Institutional Review Board of UCLA, performed in accord with the tenets of the Declaration of Helsinki. Written informed consent was obtained from each participant.

Initially, a total of 24 eyes with mild POAG, and 21 pre-perimetric glaucoma eyes were age matched with 16 eyes from normal controls. Four eyes from the POAG group and 1 eye from the pre-perimetric glaucoma group were not analyzed because of poor OCT angiography quality, leaving 56 eyes for statistical analysis.

The diagnostic criteria for glaucoma included all of the following: 1) the presence of characteristic glaucomatous optic disc damage and abnormal thinning of the circumpapillary RNFL; 2) visual field defects consistent with glaucoma, confirmed on at least two visual field examinations; 3) normal open angles on gonioscopy; and 4) no history of any other ocular or systemic diseases causing non-glaucomatous optic nerve damage. Only mild stage POAG eyes based on Hoddap-Anderson-Parrish scale [15] were included in our study as measured by visual field mean deviation scores (MD > −6.0 dB).

We included patients as pre-perimetric glaucoma who did not meet the aforementioned definition of glaucoma but had ocular hypertension (IOP > 21 mmHg) and an absence of characteristic glaucomatous optic nerve damage or detectable visual field defects [6].

The inclusion criteria for the normal subjects were defined as IOP of ≤21 mmHg, normal appearing optic nerve head, intact neuroretinal rim and normal RNFL thickness, and normal standard automated perimetry (defined as a glaucoma hemifield test within normal limits and a pattern standard deviation within 95% confidence-interval limits).

The exclusion criteria for all eyes were the following: (1) best-corrected visual acuity less than 20/40, (2) age younger than 30 years or older than 80 years, (3) refractive error greater than +3.00 diopter (D) or less than −6.00 D, (4) previous intraocular surgery except for uncomplicated cataract extraction with posterior chamber intraocular lens implantation, (5) any non-glaucomatous conditions that may cause VF loss or optic disc abnormalities, or (6) inability to perform reliably on automated VF testing. One eye from each participant was imaged and analyzed in a random manner.

All subjects were interviewed regarding their medical history. Thorough ophthalmic examinations included refractive status, slit-lamp biomicroscopy, fundus examination, IOP, central corneal thickness (CCT), and gonioscopy, performed by glaucoma specialists. The RNFL thickness, from a 3.4-mm diameter circle scan centered on the disc, was assessed with SD-OCT (The Cirrus HD-OCT (Carl Zeiss Meditec, Dublin, CA). IOP was measured using Goldmann applanation tonometry and CCT was measured with a handheld ultrasound pachymeter (Ipac Pachymeter, Reichert Ophthalmics, NY, USA). Visual field tests were performed with the Humphrey Field Analyzer II (Carl Zeiss Meditec, Inc). The system was set for the 24–2 threshold test, size III white stimulus, SITA-standard algorithm.

**Fig 1:**
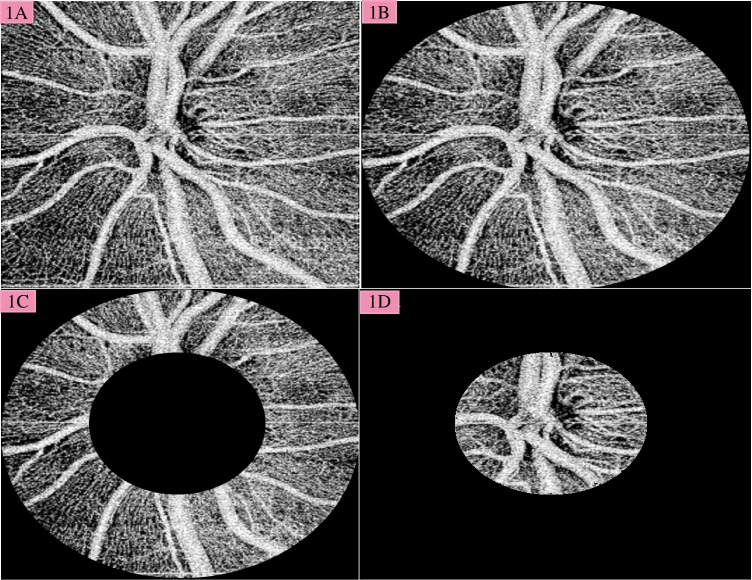
1A. A full-thickness retinal slab was selected by selecting the ILM and RPE as boundaries. The boundaries were not changed for the measurements in the disc area, and the instrument automatically connected the two ends of the RPE as outer boundary. Vessel intensity ratios from 3 regions of interest were calculated, 1B. Papillary region (3 mm circular region centered on the ONH), 1C. Peripapillary region (700 micron wide elliptical annulus centered on the disc), 1D. The optic nerve head

The optic disc region was imaged using a 3 × 3 mm scan by a 1050-nm-wavelength spectral OCT system (DRI OCT Triton, TOPCON). We used custom grading software (OCTORA) of the device to generate the maps. En-face images of the vasculature were generated from the optic nerve and retinal layers and collapsed into a single two-dimensional image set between the internal limiting membrane and retinal pigment epithelium (Fig 1). Quantitative analysis of the vessel density was performed using the publically available GNU Image Manipulation Program GIMP 2.8.14 (http://gimp.org). Average pixel density was determined from the vessels after background subtraction (Photoshop) as previously reported [16]. This was done for the entire image (Fig 1A) as well as 3 regions of interest: 1) papillary region (3 mm circular region centered on the ONH) (Fig 1B), 2) peripapillary region (700 micron wide elliptical annulus centered on the disc) (Fig 1C), and 3) the optic nerve head (Fig 1D). Vessel intensity ratios for each region of interest was calculated by dividing their average pixel density by that of the entire image. Separately, since mild glaucoma often presents with focal, rather than global defects, we divided the vessel intensity ratio for the papillary region into superior and inferior domains.

Intra-visit repeatability of the vessel densities was calculated with 2 sets of images obtained sequentially from a single visit. Variability was assessed by the coefficient of variation (CV), calculated as the root-mean-square measurement variation divided by the mean of the measured values. Inter-visit, intra-visit and inter-operator reproducibility of 2 operators were calculated from 16 normal eyes using the mean value averaged from 2 sets of images per visit.

## Statistical analysis

Data are shown as a mean value with the standard deviation. The Kruskal-Wallis test was used to analyze the significance of differences among the 3 groups and if there was a difference T test was used to check where the difference came from. Multiple linear regression analysis was used to determine the relationships between the vessel density and traditional glaucoma measurements of structure (OCT-derived RNFL thickness) and function (VF mean deviation, VF pattern standard deviation [PSD]) in glaucomatous eyes. The receiver operating characteristic (ROC) curve for the disc parameters was plotted to determine the optimum cutoff point, and area under the ROC curve (AUC) was used to determine the discrimination power between the normal and POAG. Repeatability and reproducibility were analyzed by using the CV and the ICC between measurements. The CV is the standard deviation of the measurements divided by their mean, expressed as a percentage. The ICC measures the proportion of total variability in measurements contributed by variability in measurements between different subjects, and was determined using the random-effects mixed model. The significance level was set at p < 0.05. All analyses were performed with statistical software (SPSS for Windows, version 19.0; SPSS, Inc., Chicago, IL, USA).

## Results

A total of 40 eyes from 40 patients were age-matched with 16 eyes from 16 normal controls. Kruskal Wallis testing was first performed to evaluate for overall differences among the groups. Table 1 summarized the baseline clinical characteristics of each group. No significant differences were found among these (p-values ranged from 0.421 to 0.7) except for IOP and number of glaucoma medications. As expected, the POAG patients were treated with medications, whereas the pre-perimetric glaucoma and normal eyes were not on glaucoma medications. For treatment in the POAG group, prostaglandin analogue once daily at bedtime was used in 10 eyes, while dorzolamide-timolol combination twice daily was used in 5 eyes, and brimonidine tartrate twice daily was used in 5 eyes. The duration of medication use was 3.5±2 years. Pairwise t-tests comparing POAG to suspects (p = 0.01) and POAG to normals (p = 0.09) confirmed that POAG patient had lower intraocular pressures which was consistent with the fact that they were under treatment. Use of an ocular antihypertensive eye drop or any subclass was not correlated with the vessel density measurements as determined by the Mann-Whitney U test.

**Table 1:** Characteristics of the study groups.

Variables	POAG (n: 20)	Pre-perimetric glaucoma (n:20)	Control (n:16)	P value^*^
**Age (years)**	65.375±5.2	63.13±16.43	62.2±12.43	0.7
**Gender (Male/Female)**	12/8	10/10	9/7	0.44
**Central corneal thickness (μm)**	534±16	539±29	528±21	0.6
**Ocular perfusion pressure (mmHg)**	48±6.7	50.1±3.5	49.5±3.9	0.4
**Number of IOP lowering drops**	1.8±0.5	0	0	<0.001

*Kruskal- Wallis

Mean ±Standard deviation

Overall differences were also found amongst the groups for visual field function (MD [p = 0.01] and PSD [p = 0.03]) as well as OCT-measured RNFL thickness (p = 0.004–0.04) (Table 2).

**Table 2:** Results of Diagnostic Testing.

Variables	POAG (n: 20)	Pre-perimetric glaucoma (n:20)	Control (n:16)	P value*
**Visual field mean deviation (dB)**	-1.7±1.8	-0.68±1.86	-0.35±1.6	0.01
**Visual field PSD (dB)**	2.8±1.9	1.65±2.25	1.65±1.4	0.03
**C/D**	0.63±0.17	0.58±0.12	0.3±0.1	<0.001
**Mean RNFL thickness (μm)**	79.9±11.5	88.8±11.66	97.3 ± 5.9	0.03
**Superior RNFL thickness (μm)**	94±19.9	109.56±18.86	116±16.3	0.036
**Inferior RNFL thickness (μm)**	98.5±19.04	115.66±20.35	124 ± 15.8	0.004
**Optic nerve head vessel density (%)**	70.1±7.8	78.04±7.2	86.6 ± 4.7	<0.001
**Peripapillary vessel density (%)**	80.03±5.63	86.83± 6.24	92.03± 3.95	<0.001
**Papillary area vessel density (%)**	81.6±2.7	83.86± 5	91.7±2.7	<0.001
**Superior papillary area vessel density (%)**	79.44±6.03	83.7 ± 5.3	93.4±1.96	<0.001
**Inferior papillary area vessel density (%)**	83.57±4.7	85.58 ± 3.1	90.96±2.76	<0.001

POAG = Primary Open Angle Glaucoma; PSD = pattern standard deviation; RNFL = retinal nerve fiber layer; C/D = cup/disc. Differences between groups were tested with the Kruskal-Wallis test. Mean ±Standard deviation

*The mean difference is significant at the 0.05

Pairwise t-tests showing a statistically significant difference between POAG and pre-perimetric glaucoma patients (p = 0.08) but not between controls and suspects (p = 0.6–1.0) for visual field performance suggested that the overall difference found came from worse visual field scores in POAG patients (Table 3). However, pairwise t-tests with statistically significant differences between both POAG vs suspects (p = 0.02) and controls vs suspects (p = 0.012) for mean RNFL thickness, help confirm that structural loss often precedes perimetric loss (since pre-perimetric glaucoma eyes had lower RNFL thickness compared to normal) (Table 3). There was no otherwise statistically significant difference found for VF MD, VF PSD, and C/D area ratio between the normal and pre-perimetric glaucoma patients (Table 3).

**Table 3:** Pairwise comparisons to distinguish the “suspects” from POAG and healthy controls.

Variables	Control (n:16)	Pre-perimetric glaucoma (n:20)	P value*	POAG (n: 20)	Pre-perimetric glaucoma (n:20)	P value*
**Visual field mean deviation (dB)**	-0.35±1.6	-0.68±1.86	0.6	-1.7±1.8	-0.68±1.86	0.08
**Visual field PSD (dB)**	1.65±1.4	1.65±2.25	1.0	2.8±1.9	1.65±2.25	0.08
**C/D**	0.3±0.1	0.58±0.12	0.0001	0.63±0.17	0.58±0.12	0.29
**Mean RNFL thickness (μm)**	97.3 ± 5.9	88.8±11.66	0.012	79.9±11.5	88.8±11.66	0.02
**Optic nerve head vessel density (%)**	86.6 ± 4.7	78.04±7.2	0.0002	70.1±7.8	78.04±7.2	0.002
**Peripapillary vessel density (%)**	92.03± 3.5	86.83± 6.24	0.007	80.03±5.63	86.83± 6.24	0.001
**Papillary area vessel density (%)**	91.7±2.7	83.86± 5	0.001	81.6±2.7	83.86± 5	0.083
**Superior papillary area vessel density (%)**	93.4±1.96	83.7 ± 5.3	0.001	79.44±6.03	83.7 ± 5.3	0.023
**Inferior papillary area vessel density (%)**	90.96±2.76	85.58 ± 3.1	0.001	81.57±4.7	85.58 ± 3.1	0.003

POAG = Primary Open Angle Glaucoma; PSD = pattern standard deviation; RNFL = retinal nerve fiber layer; C/D = cup/disc. Differences between groups were tested with the Independent t test. Mean ±Standard deviation

*The mean difference is significant at the 0.05

In OCTA-measured vessel density, overall differences were found between the groups for optic nerve head, papillary, and peripapillary vessel density (all p<0.001, Table 2). Like for OCT RNFL, pairwise t-tests showed that for all vessel density measures (except papillary area vessel density comparing POAG to suspects) that POAG to suspects (p = 0.003–0.023) and controls to suspects (p = 0.001–0.007) were statistically significantly different with the suspects having values in between those of POAG and normal controls (Table 3). For intra-visit, inter-visit and inter-observer repeatability, the ICC values were based on measurements from 16 normal subjects (Table 4).

**Table 4:** Repeatability measurements with healthy eyes.

	Intervisit ICC (95% CI)	Intravisit ICC (95% CI)	Interobserver ICC (95% CI)
**Peripapillary area vessel density**	0.927 (0.896–0.949)	0.953 (0.933–0.968)	0.977 (0.966–0.984)
**Optic nerve head vessel density**	0.905 (0.865–0.933)	0.951 (0.930–0.966)	0.970 (0.957–0.979)
**Papillary area vessel density**	0.934 (0.906–0.954)	0.955 (0.935–0.969)	0.964 (0.948–0.975)

ICC: Intraclass Correlation Coefficient, CI: Confidence Interval).

In the POAG group, the univariate regression analysis using the Pearson correlation coefficient showed that optic nerve head vessel density was significantly correlated with VF MD (r = 0.829, p<0.001), VF PSD (r = 0.47, p = 0.04), and RNFL thickness (r = 0.512, p = 0.04). Also superior papillary area vessel density was significantly correlated with superior RNFL thickness (r = 0.436, p = 0.02), inferior papillary area vessel density was significantly correlated with inferior RNFL thickness (r = 0.523, p = 0.04).

The areas under the receiver operating characteristic curve (ROC) for differentiating normal from POAG was 0.956 for peripapillary vessel density, 0.931 for optic nerve head vessel density and 0.956 for papillary area vessel density respectively (Table 5).

**Table 5:** Variables area under the curve comparison in study groups.

	Control vs POAG	Control vs Pre-perimetric Glaucoma
**Peripapillary area vessel density**	0.956 (0.883–1.000) *p*<0.001	0.756 (0.566–0.946) *P* = 0.03
**Optic nerve head vessel density**	0.931 (0.838–1.000) *p*<0.001	0.863 (0.720–1.000) *P* = 0.02
**Papillary area vessel density**	0.956 (0.883–1.000) *p*<0.001	0.956 (0.887–1.000) *P* = 0.001
**Superior papillary area vessel density**	1.000 (1.000–1.000) *p*<0.001	0.981 (0.938–1.000) *p*<0.001
**Inferior papillary area vessel density**	0.9 (0.762–1.000) *P =* 0.001	0.819 (0.659–0.979) *P* = 0.007

Data are area under the curve (95% confidence interval). Null hypothesis: true area = 0.5

The area under the ROC for differentiating normal and pre-perimetric glaucoma eyes was 0.956 for papillary area vessel density. The ROC curves showed that the cutoff point was 91.3% for peripapillary vessel density (94% sensitivity), 86.5% for optic nerve head vessel density (94% sensitivity) and 92.15% for papillary area vessel density (100% sensitivity) between the controls and POAG eyes at 95% specificity values (Table 6).

**Table 6:** Cutoff points for the peripapillary area vessel density, optic nerve head vessel density, papillary area vessel density, superior and inferior papillary area vessel density with the sensitivity values at 95% specificity.

	POAG		Pre-perimetric glaucoma	
	Cut off point	Sensitivity	Cut off Point	Sensitivity
**Peripapillary area vessel density**	91.3	94	92.5	87.5
**Optic nerve head vessel density**	86.5	94	85.4	93.8
**Papillary area vessel density**	92.15	100	91.75	100
**Superior papillary area vessel density**	93.8	100	93.85	100
**Inferior papillary area vessel density**	90.65	93.8	91.4	81.3

Data derived from ROC curve.

We also performed the analysis of the area under the ROC for glaucoma and suspect eyes and it was 0.778 (p = 0.06) for peripapillary vessel density, 0.789 (p = 0.05) for optic nerve head vessel density. Since the p-values are almost statistically significant, it is possible that expanding the study cohort to include more eyes might reveal that optic nerve head vessel density can differentiate pre-perimetric glaucoma from perimetric glaucoma.

Area under the ROC for mean RNFL in POAG and controls, POAG and suspects, suspects and controls were 0.916 (p< 0.0001), 0.711 (p = 0.042) and 0.772 (p = 0.022) respectively. This result reveals that RNFL thickness is a good parameter with high sensitivity and specificity values for differentiating glaucoma from pre-perimetric glaucoma and normal eyes.

In the POAG group, multiple linear regression analysis in which the vessel density was considered as the dependent variable, was performed. RNFL thickness was found as a predictor of optic nerve head vessel density. Age, IOP, VF MD, VF PSD and C/D area ratio were not significant explanatory variables when grouped with RNFL thickness in the multivariate models. This showed that optic nerve head vessel density in POAG group were more strongly linked to RNFL thickness than to any other variables (p = 0.65 for peripapillary vessel density, p = 0.04 for optic nerve head vessel density and p = 0.112 for papillary area vessel density).

## Discussion

In our current study using OCT angiography, we were able to demonstrate lower retinal vessel densities for eyes with mild glaucoma compared with normal eyes, as well as, compared with pre-perimetric glaucoma eyes. In addition, one of the most revealing findings in our study is that we were able to demonstrate lower retinal vessel densities in our pre-perimetric glaucoma group (which includes patients with essentially normal perimetry testing but mild reduction in OCT-derived RNFL thickness measurements) compared with normal eyes. Thus, we were able to distinguish patients with pre-perimetric glaucoma from age-matched normal eyes using OCTA-derived vessel density measurements, and found correlation with mild reduction in OCT-derived RNFL thickness measurements. This may have important implications in increasing our understanding of the pathophysiology of glaucoma and its relationship with retinal vasculature, as previously suggested and in agreement with previous studies [10–13]. Furthermore, having novel structural parameters such as OCTA-derived retinal vascular measurements, in addition to the currently used RNFL and neuro-retinal rim thickness measurements, may enhance the clinician’s ability to detect early/mild glaucoma–which can often be challenging in the clinical setting, especially in cases with normal perimetry testing.

We defined functional loss in our study as seen in standard automated perimetry which is widely used in the clinical setting and it has been shown previously that structural parameters such as loss of RNFL thickness can be typically identified before perimetric loss is detected since structural loss can precede functional loss by years [17,18]. A lower vessel density found in our cases of early POAG and even pre-perimetric glaucoma eyes suggests that the retinal vasculature attenuation may start early in the course of glaucomatous disease cascade. Previously, Kerr et al [19] reported that patients with untreated POAG had a reduction in lamina cribrosa and temporal neuroretinal rim blood flow compared to patients with ocular hypertension. They also indicated that reduced ONH blood flow may be an early event in glaucoma which is consistent with many studies [11,20]. Additionally, Pareira et al [20] evaluated retinal vessel density in a 3.46 mm circle with scanning laser ophthalmoscope from a Fourier domain OCT and concluded that vessel density may have a clinically relevant influence on the RNFL distribution. Thus, it is possible that retinal vessel attenuation may become a parameter that is particularly useful for detection of early glaucomatous disease. Future studies that include more advanced levels of glaucoma and longitudinal data analyses are needed to determine whether vessel density continues to be correlated to functional loss.

There is evidence that altered optic nerve head blood flow may play a role in the development and progression of glaucoma. A large number of clinical studies have been performed to evaluate this issue by using different techniques for the assessment of ocular blood flow [19–22].

Previously, fluorescein angiography (FA) has been demonstrated to evaluate perfusion defects of the optic disc of patients with glaucoma and were correlated with visual field alterations [19]. FA, however, is not commonly used for detection and monitoring of glaucoma because of its invasive nature and the difficulty in quantification. Scanning laser doppler flowmetry was used in a study [21] to investigate the ocular hemodynamic effects of patients with glaucoma or ocular hypertension and there was no significant difference in ocular blood flow parameters between patients with POAG and those with ocular hypertension. Laser doppler flowmetry and laser speckle flowgraphy, which are noninvasive techniques, have also been reported as measures of disc perfusion. In a previous study, laser doppler flowmetry showed no significant difference in blood flow parameters of the optic nerve head between POAG patients and glaucoma suspects. However, in the same study, blood flow in the optic cup, superior-temporal rim and the inferior-temporal rim was found to be significantly lower in glaucoma suspects than in healthy control subjects [11]. Laser speckle flowgraphy is a noninvasive instrument that has been used to assess intraocular circulation and was also able to show a reduction in the microcirculation of the optic nerve head of glaucoma patients [20]. However at current scan speeds, OCT angiography is not able to measure actual blood flow, but it can measure the caliber of vessels, suggesting that the angiography-based measurements mainly gives information about the disc and retinal microvasculature. Using OCTA, our study did find statistically significant differences in vessel density between glaucoma suspect and normal control groups. Previously, OCT angiography on a swept-source system reported the ability to differentiate glaucomatous eyes from normal eyes based on quantitative analysis of the findings [6]. Some OCT angiography studies found a decrease in the disc / peripapillary flow index and vessel density in the glaucomatous eyes, which was correlated with the severity of glaucoma damage. It was concluded that OCT angiography disc blood flow calculations showed excellent correlation with the severity of glaucoma [12,13]. Wang et al reported that altered flow index and vessel density values may be good indicators of eyes with severe open angle glaucoma [12]. Our study adds to these findings since our results showed that vessel density changes may be seen even in very early phases of glaucomatous disease.

The current study’s results of lower vessel density values in glaucoma vs age-matched normal eyes are in agreement with previous swept-source OCT studies [6, 12, 13] and with previous studies [19,10]. Additionally, a statistically significant difference was found between POAG and pre-perimetric glaucoma groups for peripapillary and optic nerve head vessel density. Therefore, structural vascular changes may play an important role in POAG pathophysiology. Further, vessel density was found to be closely correlated to the structural loss and visual field loss in POAG eyes. The multiple linear regression analysis was performed in POAG group and it was found that optic nerve head vessel density in POAG group was more strongly linked to RNFL thickness than to any other variables. The close correlation between the optic nerve head vessel density and visual field MD and PSD, and OCT mean RNFL thickness suggests that OCT angiography needs to be investigated for correlation with possible progression of glaucoma. Additionally, the peripapillary, optic nerve head and papillary area vessel density was lower in pre-perimetric glaucoma group than in control group, showing that vessel density measurements may have the potential to be used for differentiating suspect eyes from healthy eyes.

Previous OCT angiography reports showed that flow index and vessel density decreased in glaucomatous eyes [12,13] with different OCT technologies than that employed in our study. In fact, similar results between different OCT angiography devices gives more support to our results examining retinal vascular changes in the cascade of glaucoma.

In our study, we compared sensitivity at 95% specificity value to find out if the variables deviate significantly from a normal distribution. The specificity for papillary area vessel density and mean RNFL thickness of POAG eyes was fixed at the same level (95%), while the sensitivity for papillary area vessel density was high (100%) and same as the mean RNFL thickness (100%). So the papillary area vessel density may be used as a potentially reliable diagnostic parameter to differentiate normal eyes from POAG eyes. Area under the ROC curve for differentiating normal and POAG eyes for papillary area vessel density (0.956 (p<0.001)) showed that this parameter may have potential to be used in challenging conditions for glaucoma diagnosis. In agreement with other OCT angiography studies [6,12,13], our current study also had high intra-visit repeatability and high inter-visit reproducibility.

Even though we found a strong correlation between reduced RNFL thickness and attenuated retinal vascular density, there were significant limitations to our study. One important limitation was that we were not able to measure retinal blood flow index; this was due to a software limitation of our OCT device. Additionally, this software had a projection artifact from superficial blood vessels to deeper tissue levels which prevented us from separately measuring superficial and deep ONH vessel density. As OCT angiography technology evolves with automated calculation of retinal blood flow, it would be useful to correlate retinal vessel density to retinal blood flow. Optic nerve head area does not have a basement membrane, and cupping varies significantly between normal individuals and glaucoma patients. To evaluate this area specifically, it would be useful to measure prelaminar vessel density in future studies with more advanced software. Another important limitation of our study is that we cannot rule out the effect of glaucoma and blood pressure medications on our measurements. Most patients in our glaucoma group were receiving multiple ocular antihypertensive eye drops. In our statistical analyses of our sample, use of an ocular antihypertensive eye drop or any subclass was not correlated with the vessel density measurements but our study may not have been powered to detect such a difference. Therefore, it is not possible to determine their individual effects on vessel density with our small sample size, and we cannot entirely rule out the possibility that the glaucoma drops could somehow be responsible for the vascular changes. We consider this less likely because glaucoma medications were previously reported to have been no significant effect [23]. We are planning to perform a study in the future to remove the confounding effects of the drugs. In addition, although our results noted statistically significant differences between groups, our sample size was not very large and additional patient recruitment will likely address this shortcoming. All these measurements were taken on initial visits with the patient before treatment was initiated, if any. The current study is cross-sectional and needs longitudinal follow-up to further assess associations over time. Furthermore, we included both large and small vessel measurements in our data analysis, and thus cannot comment on the relative contributions of micro- vs. macro-vessels towards their individual contributions to glaucoma.

## Conclusion

To the best of our knowledge, our study is the first to report that retinal blood vessel density measures derived using non-invasive OCT angiography showed a stepwise decrease from normal eyes to pre-perimetric glaucoma eyes to mild POAG eyes. Furthermore, this difference in vessel density was seen in all three of the anatomic sites measured: optic nerve head, papillary, and peripapillary regions. Importantly, this difference was seen even though the POAG group in our study consisted only of mild disease, and lower retinal vessel density was also noted in our pre-perimetric glaucoma group. This shows that OCT angiography may provide new structural parameters that could potentially be used by clinicians to diagnose glaucoma at earlier stages. Our data suggest that retinal vascular changes may develop early in the glaucomatous process and may not develop solely as a result of advanced glaucoma damage. Even though the importance of retinal blood flow in glaucoma has been reported in prior research studies [10–24], the inability to get easily acquired measurement values has limited the wide-spread utility and applicability of these parameters in a clinical setting until now. Reduced retinal vessel density and/or retinal blood flow measurements using the evolving OCT angiography techniques may provide additional parameters that can be utilized in a clinical setting for glaucoma diagnosis and management.

## Supporting information

S1 Table Summary data and statistics of the study. (XLSX)
